# Overcoming inherent resistance to histone deacetylase inhibitors in multiple myeloma cells by targeting pathways integral to the actin cytoskeleton

**DOI:** 10.1038/cddis.2014.98

**Published:** 2014-03-20

**Authors:** S Mithraprabhu, T Khong, A Spencer

**Affiliations:** 1 Myeloma Research Group, Australian Centre for Blood Diseases, The Alfred Hospital/Monash University, Melbourne, Australia; 2 Malignant Hematology and Stem Cell Transplantation, The Alfred Hospital, Melbourne, Australia; 3 Department of Clinical Hematology, Monash University, Clayton, Australia

**Keywords:** histone deacetylase inhibitors, resistance, actin cytoskeleton pathway, multiple myeloma

## Abstract

Histone deacetylase inhibitors (HDACi) are novel chemotherapeutics undergoing evaluation in clinical trials for the potential treatment of patients with multiple myeloma (MM). Although HDACi have demonstrable synergy when combined with proteasome inhibitors (PIs), recent evidence indicates that combination of HDACi and PI is beneficial only in a subset of patients with advanced MM, clearly indicating that other rational combinations should be explored. In this context we hypothesized that understanding the molecular signature associated with inherent resistance to HDACi would provide a basis for the identification of therapeutic combinations with improved clinical efficacy. Using human myeloma cell lines (HMCL) categorized as sensitive, intermediate or resistant to HDACi, gene expression profiling (GEP) and gene ontology enrichment analyses were performed to determine if a genetic signature associated with inherent resistance to HDACi-resistance could be identified. Correlation of GEP to increasing or decreasing sensitivity to HDACi indicated a unique 35-gene signature that was significantly enriched for two pathways – regulation of actin cytoskeleton and protein processing in endoplasmic reticulum. When HMCL and primary MM samples were treated with a combination of HDACi and agents targeting the signaling pathways integral to the actin cytoskeleton, synergistic cell death was observed in all instances, thus providing a rationale for combining these agents with HDACi for the treatment of MM to overcome resistance. This report validates a molecular approach for the identification of HDACi partner drugs and provides an experimental framework for the identification of novel therapeutic combinations for anti-MM treatment.

Multiple myeloma (MM) is an incurable B-cell neoplasm characterized by drug resistance and complex cytogenetic abnormalities with recent genome-wide sequencing data clearly indicating that there is no single genetic lesion underlying myelomagenesis that could be targeted therapeutically.^1, 2^ With the adoption of high-dose chemotherapeutic strategies and the emergence of novel therapeutics (immunomodulatory drugs and proteasome inhibitors (PIs)) over the past 15 years median survival, although improved, remains <10 years. Therefore, there is an ongoing need to identify more effective novel therapeutic approaches for MM through determination of factors that contribute to inherent resistance of MM cells.

Histone deacetylase inhibitors (HDACi) are epigenetic modifying agents that initiate a myriad of cellular responses impeding both the proliferation and viability of MM cells. HDACi are currently under evaluation in Phase II and III clinical trials in combination with approved and investigational agents for MM. PIs are known to synergize effectively with HDACi in MM cells and the observed synergism is postulated to occur by simultaneously inhibiting the aggresome pathway, through increased acetylation of the HDAC6-substrate tubulin, and the proteasome pathway, leading to accumulation of polyubiquitinated proteins, and subsequent apoptosis.^3, 4^ A recently reported clinical trial combining the HDACi vorinostat and bortezomib demonstrated that a subset of bortezomib-refractory MM patients clearly benefited from this combination,^5^ whereas a randomized Phase III trial of patients with less advanced disease comparing the same combination with bortezomib alone, demonstrated a disappointingly modest advantage to the former.^6^ This raises the notion that in the context of such a genetically heterogeneous disease and the availability of broadly effective anti-MM therapies, at least in the early stages of the disease, that the current drug development paradigm whereby newer potentially effective MM agents are simply added to ‘standards of care' and tested in unselected MM patient populations may be unlikely to identify agents, that if utilized in a more rational and selected fashion, may be highly effective. Therefore, the identification of genetic factors intrinsic to MM cells that mediate differences in response to HDACi would facilitate both the selection of appropriate patients for therapy with HDACi and the resulting improvement in treatment outcome.

A range of other developmental therapeutics that have undergone *in vitro* evaluation in combination with HDACi and have demonstrated some degree of synergy in a limited range of human myeloma cell lines (HMCL) include MAPK (mitogen-activated protein kinase)/ERK (extracellular signal regulated kinases) inhibitors,^7, 8^ HSP90 (heat shock protein 90) inhibitors,^9, 10^ mTOR (mammalian target of rapamycin) inhibitors,^11^ B-cell lymphoma 2 (Bcl-2) inhibitors,^12, 13^ DNA damage-inducing agents^14^ and TRAIL (TNF-related apoptosis-inducing ligand) inhibitors.^15, 16^ These partner drugs have been chosen based on current clinical availability (PIs and DNA damage inducing agents) or *in vitro* observations of pathway regulation following exposure to HDACi resulting in acquired resistance (NF-KB (nuclear factor kappa-light-chain-enhancer of activated B cells), MEK/ERK, Bcl-2 inhibitors). However, a comprehensive analysis of the molecular determinants of HDACi responsiveness that could optimize HDACi partner drug selection has never been undertaken.

Microarray-based technologies for genome-wide screening of gene expression have increased the prospects of better understanding molecular determinants of drug responsiveness. In this report, microarray-based basal mRNA expression profiles of HDACi-resistant, intermediate and sensitive HMCL were compared utilizing bioinformatics approaches to identify pathways associated with inherent resistance to HDACi. Genes belonging to two pathways – regulation of actin cytoskeleton and protein processing in endoplasmic reticulum were enriched in the differentially regulated gene sets. We hypothesized that a combination of HDACi and inhibitors that are known to target pathways integral to the actin cytoskeleton should induce synergistic cell death. Combining HDACi with a range of diverse inhibitors targeting these pathways induced synergistic killing of MM cells thus validating the approach. These data provide a rationale for the clinical evaluation of these combinations and support the further exploration of microarray-based approaches for the identification of other novel anti-MM drug combinations.

## Results

### HMCL have differential responses to HDACi

**Figure 1:**
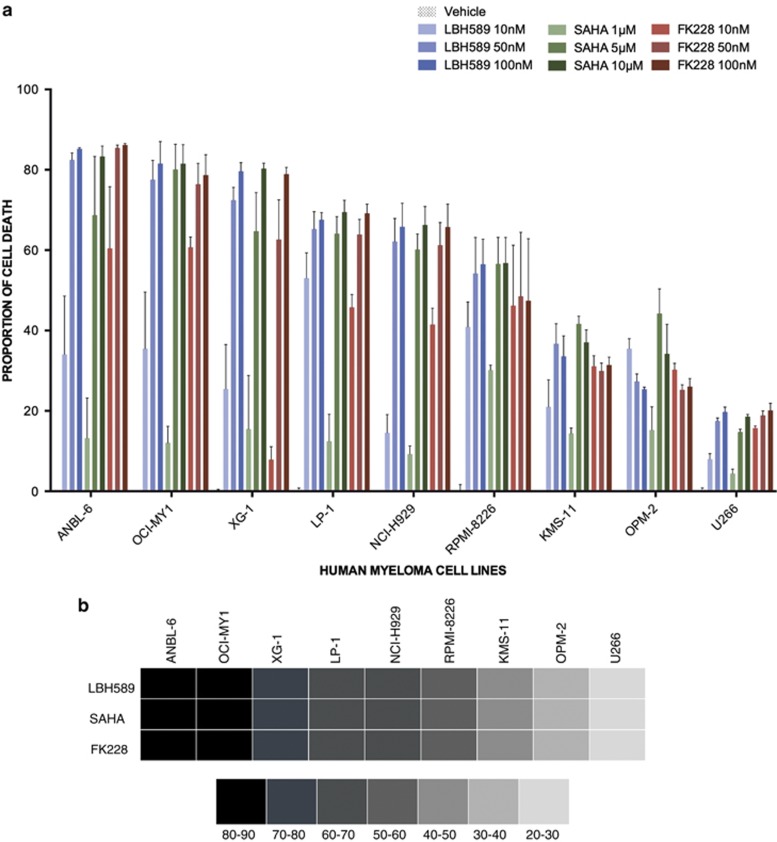
Determination of sensitivity of HMCL to HDACi LBH589, SAHA and FK228. (**a**) Nine HMCL were exposed to three doses of HDACi (LBH589 and FK228 at 10, 50 and 100 nM and SAHA at 1, 5 and 10 *μ*M) for 48 h and the proportion of cell death determined through flow cytometric enumeration of propidium iodide staining. Cell death is normalized to proportion of cell death in untreated samples. Data are represented as mean±S.E.M. from three experiments. (**b**) Representation of cell death induced by LBH589 (100 nM), SAHA (10 *μ*M) and FK228 (100 nM) in nine HMCL. Mean cell death induced by each HDACi is represented. Different shades represent the amount of cell death as shown in the boxes.

The HMCL chosen for this study reflect the heterogeneous nature of MM, with 3/9 (OPM2, NCI-H929 and LP-1) harboring *t*^4, 14^, 3/9 (ANBL-6, RPMI-8226 and OCI-MY1) *t*^14, 16^, 2/9 (XG-1 and U266) *t*^11, 14^ and 1/9 (KMS11) bearing both *t*^4, 14^ and *t*^14, 16^ translocations, in addition to secondary genetic lesions found within each HMCL.^17, 18, 19, 20^ HDACi that are currently being evaluated in Phase II and III MM clinical trials were chosen (LBH589 – Panobinostat, SAHA – Vorinostat and FK228 – Depsipeptide). An initial pre-screen of six HMCL (U266, OPM2, KMS11, RPMI-8226, NCI-H929 and OCI-MY1) was performed with increasing concentrations of LBH589 and SAHA to determine the effect on proliferation using methyl tetrazolium salt assay (Supplementary file 2A, B). HDACi were able to induce varying levels of proliferation inhibition in HMCL, with U266 being the most resistant, followed by OPM2. To determine if this pattern of response could be re-capitulated in a cell death assay, a larger cohort of nine HMCL was treated with three HDACi (two pan-inhibitors and one class I specific inhibitor). HMCL were treated with increasing doses of LBH589 (10, 50 and 100 nM), FK228 (10, 50 and 100 nM) and SAHA (1, 5 and 10 *μ*M) for 48 h and the proportion of cell death was assessed using propidium iodide staining (Figure 1a). The mean proportion of cell death caused by LBH589 (100 nM), SAHA (10 *μ*M) and FK228 (100 nM) was calculated and five HMCL (ANBL6, OCI-MY1, XG-1, LP-1 and NCI-H929) were categorized as sensitive (60–90% cell death), two (RPMI-8226 and KMS-11) as intermediate (30–60% cell death) and two (OPM2 and U266) as resistant (10–30% cell death) (Figure 1b). The proportion of cell death induced by the three inhibitors was similar in all HMCL tested indicating that cell death induced by HDACi, whether a pan-HDACi or a Class I specific inhibitor, is orchestrated by similar mechanism(s). It has also recently been shown that cell death induced by these inhibitors is primarily through the modulation of class I HDAC.^21^ To determine the factors that might contribute to resistance, HDACi-induced cell death was correlated to the known genetic lesions harbored by the HMCL and to the levels of HMCL HDAC expression (Class I, II and IV HDAC comprising of HDAC 1-11). The response to HDACi did not correlate with translocation status, common genetic lesions (available through the cosmic database http://cancer.sanger.ac.uk/cancergenome/projects/cosmic/), including BIRC2/3 (baculoviral IAP repeat-containing protein 2/3), BRAF (v-Raf murine sarcoma viral oncogene homolog B1), CDKN2C (cyclin-dependent kinase inhibitor 2C), FGFR3 (fibroblast growth factor receptor 3), KRAS (v-Ki-ras2 Kirsten rat sarcoma viral oncogene homolog), NRAS (neuroblastoma RAS viral oncogene homolog), RB1 (retinoblastoma protein), TP53 (phosphoprotein p53) and TRAF3 or HDAC expression levels (data not shown). In order to ensure that HDACi-resistant HMCL were not resistant to other conventional and novel anti-MM agents because of an inherent inability of some HMCL to execute cell death, comparison of the cell death profile of the HMCL following HDACi treatment was compared with that following treatment with various alternative anti-MM agents. Among the anti-MM agents tested, only carfilzomib (data not shown) had a cell death profile similar to HDACi, with OPM2 and U266 being resistant, whereas other agents including inhibitors of signaling pathways (JAK-STAT (janus kinase/signal transducers and activators of transcription)),^22^ of beta-catenin, notch signaling (unpublished results) and of Class III HDAC (Sirtuins)^23^ had quite distinctive effects on HMCL dissimilar to those induced by HDACi.

### A 97 gene signature is associated with resistance to HDACi

**Figure 2:**
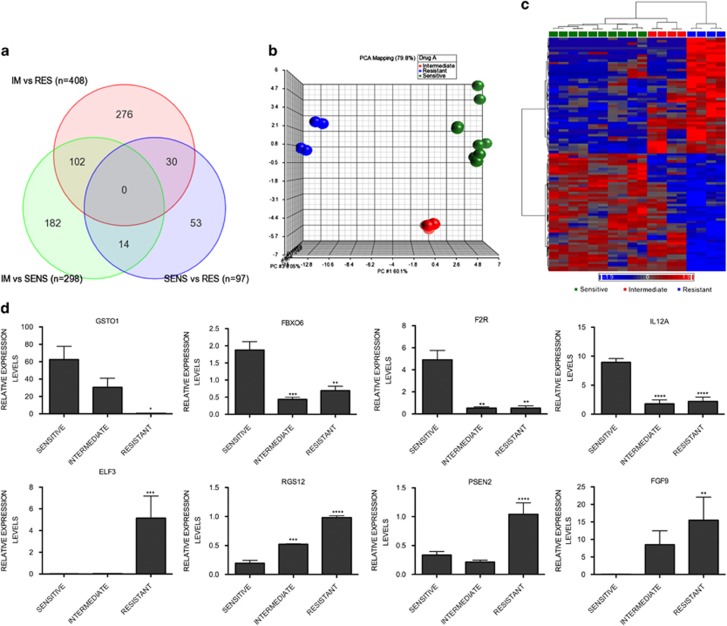
Genetic signature associated with resistance to HDACi. (**a**) VENN diagram of genes that are differentially regulated in the sensitive (SENS) *versus* resistant (RES), intermediate (IM) *versus* SENS and IM *versus* RES. Differential expression was defined as probes associated with *P*-values <0.05, and fold changes >1.5. (**b**) Unsupervised PCA of sensitive, intermediate and resistant cell lines indicating correlation of gene expression to HDACi responsiveness. Resistant HMCL (red) clustered together, whereas intermediate HMCL (green) had a profile closer to that of the sensitive HMCL (blue). (**c**) Heat map representation of the 97 genes differentially regulated between the SENS *versus* RES cell lines. Heat map also includes the expression profile of 97 genes in the intermediate cell lines indicating a GEP overlapping with both sensitive and resistant cell lines. (**d**) Eight genes were selected for validation of the Illumina HT-12 microarray. Column graphs show relative expression levels of the genes as mean±S.E.M. of sensitive (*n*=5), intermediate (*n*=2) and resistant (*n*=2) cell lines. GraphPad prism was utilized to determine significant differences between each cohort (*P<0.05, **P<0.01, ***P<0.001, ****P<0.0001)

Since neither translocation nor HDAC gene expression levels correlated with cell death, we hypothesized that inherent resistance of a HMCL to HDACi must be multi-factorial. In order to determine what signaling pathways might be associated with HDACi-resistance, RNA from HMCL was extracted and Illumina HT-12 microarray was performed to determine the gene expression profile (GEP) of the sensitive, intermediate and resistant HMCL. Differential expression of probes between the three categories was determined and a VENN diagram representation of probes that were significantly different between sensitive and resistant (*n*=97), intermediate and resistant (*n*=408) and intermediate and sensitive (*n*=298) HMCL groups is shown (Figure 2a). Unsupervised principle component analysis (PCA), which separated samples according to their HDACi-responsiveness (fold change >2, false discovery rate *q* value of *q* <0.05) indicated that the resistant HMCL clustered together with a distinct genetic signature and the intermediate HMCL had a profile similar to that of sensitive HMCL (Figure 2b). Further analysis was performed on the probe set (*n*=97 probes) that was significantly different. The list of genes that were differentially regulated between the sensitive and resistant HMCL groups is provided in Supplementary file 3. Similar to the PCA, a heat map representation of these probes indicated that intermediate HMCL had a GEP overlapping with both the sensitive and resistant HMCL (Figure 2c). Gene ontology (GO) enrichment analysis using the KEGG databases indicated that this cohort of 97 genes was enriched for seven pathways (Supplementary file 3). Pathways included other types of O-glycan biosynthesis (*P*=0.01), notch signaling pathway (*P*=0.01), glutathione metabolism (*P*=0.01), protein processing in endoplasmic reticulum (*P*=0.02), melanoma (*P*=0.02), apoptosis (*P*=0.03) and regulation of actin cytoskeleton (*P*=0.03). A number of biological processes, molecular functions and cellular components were also significantly enriched for in the differentially regulated gene set (Supplementary file 3). Validation of the microarray was performed through quantitative reverse transcriptase-polymerase chain reaction (RT-PCR) assessment of select genes including *FGF9* (fibroblast growth factor 9), *ELF3* (E74-like factor 3), *RGS12* (regulator of G-protein signaling 12), *PSEN2* (presenilin 2), *IL12A* (interleukin 12A), *GSTO1* (glutathione S-transferase omega-1), *FBXO6* (F-box protein 6) and *F2R* (F2R) (Figure 2d).

### A 35-gene signature correlates with the degree of sensitivity to HDACi

**Figure 3:**
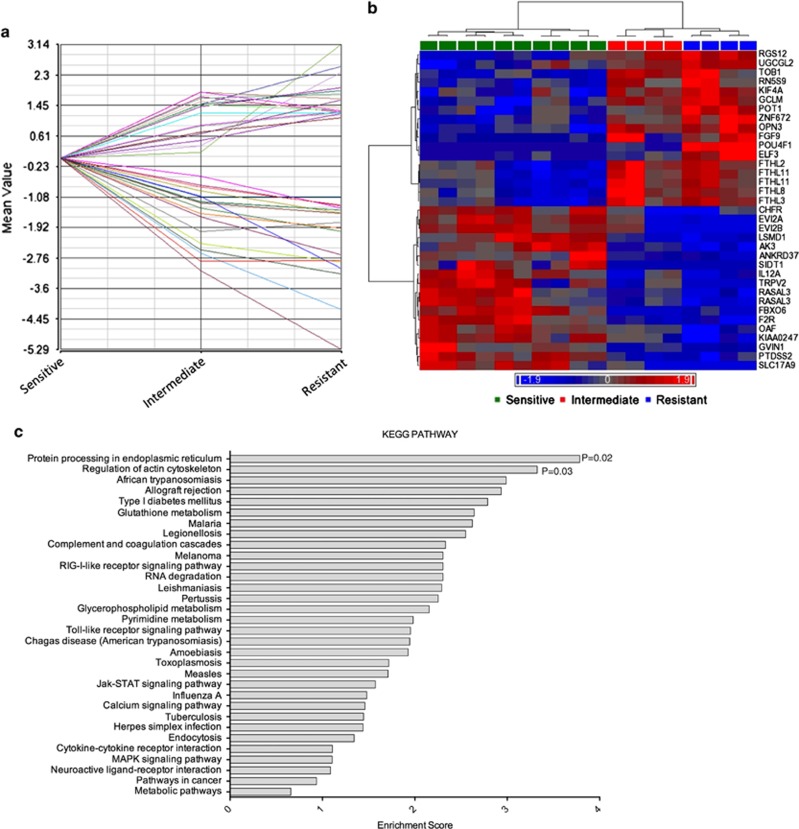
A 35-probe signature correlates to grade of sensitivity to HDACi. (**a**) Probes were tested for their correlation to grade of sensitivity using Spearman rank algorithm. Correlation plot indicating greater than 80% positive or negative correlation of gene expression in resistant and intermediate compared with sensitive HMCL. (**b**) Heat map representation of 35 probes with increasing or decreasing gene expression levels in sensitive, intermediate and resistant HMCL. (**c**) Graph representing the enrichment score for pathways that the differentially regulated gene set was enriched for. A score >1 indicates an overexpression of the functional group, whereas a score >3 indicates a significant overexpression of the functional group. Genes associated with the regulation of actin cytoskeleton (*P*=0.03) and protein processing in endoplasmic reticulum (*P*=0.02) were significantly enriched in the gene set

**Figure 4:**
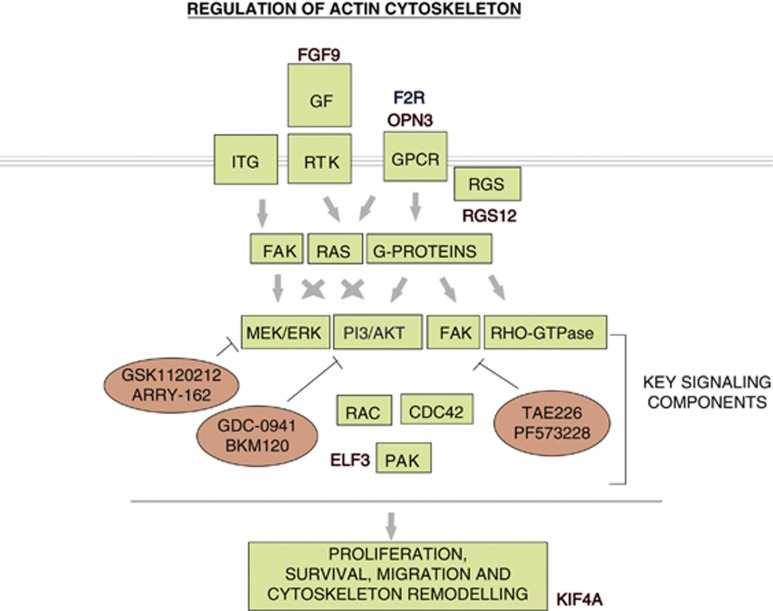
Simplified representation of the regulation of actin cytoskeleton pathway and differentially regulated genes. Regulation of actin cytoskeleton encompasses signaling to the cytoskeleton through GPCRs, ITGs and growth factor (GF) – RTKs, leading to diverse cell functions including changes in cell motility, proliferation and survival.^24, 25, 26, 27^ GPCR signaling is activated by a number of external stimuli and signals through heterotrimeric G-proteins, which in turn stimulates a variety of downstream signaling pathways including MAPK, PI3K, FAK and Rho family of GTPases (Rho, Rac and cell division cycle 42 – CDC42) and downstream protein kinase effectors including p21-activated kinase (PAK).^26, 27^ Within the 35-gene signature, F2R and OPN3, both GPCR proteins, were dysregulated. Regulators of G-protein signaling (RGS) are multifunctional signaling proteins that directly bind to activated G-proteins and are integral for modulation of the GPCR signaling process. RGS12, a RGS protein, is upregulated in the resistant cell lines. RTKs are high-affinity cell surface receptors for GF and are key regulators of normal cellular processes and progression of many types of cancers. Predominant signaling pathway that mediates signals from RTK-GF is the MAPK pathway. FAK and PI3K pathways are also known to have important roles in signal transduction. FGF9, a GF, is upregulated in the resistant cell lines. ITGs are a family of cell-surface-adhesion receptors that transmit signals to the cell to determine migration, survival, differentiation and motility in context with the GPCR and RTK-GF signaling. One of the first integrin signaling molecules to be activated is FAK, which in turn activates other signaling pathways. Signaling initiated by GPCR, RTK-GF or ITGs results in remodeling of the actin cytoskeleton and KIF4A, upregulated in resistant cell lines, is a known actin cytoskeleton protein. Genes upregulated in the resistant cell lines are shown in red, whereas downregulated genes are shown in blue

The GEP of intermediate HMCL had a signature that overlapped with both sensitive and resistant GEP (Figure 2c). Therefore, we hypothesized that there may be a genetic signature that correlated with increasing or decreasing sensitivity to HDACi. Hence, an assessment, independent of the initial analysis that identified the 97 genes was performed for all probes using Spearman's rank algorithm. The Spearman's coefficient (*r*_s_) that quantifies the strength of the association between the HMCL responsiveness to HDACi and gene expression levels was determined. HDACi response was set at arbitrary values of 1 (sensitive), 5 (intermediate) or 10 (resistant) and the mean signal intensity (*y*-axis) of each probe was normalized to levels in the sensitive HMCL (Figure 3a). An absolute and stringent correlation of 0.8 was utilized and as a result, among all the probes tested, only 4889 probes passed the variance filter and despite the genetic heterogeneity of the HMCL, it was striking to note that 35 genes had a positive or negative correlation of >0.8 with increasing resistance to HDACi. Although relaxing the correlation coefficient filter delivered more genes and possibly more significance, it did not have a conclusive trend of expression from low to high or high to low over the three groups. Therefore, the more stringent correlation was set to identify genes with correlation to resistance. A heat map representation of these probes is shown (Figure 3b). GO enrichment analysis indicated that a number of biological processes, molecular functions and cellular components were significantly enriched for in the differentially regulated gene set (Supplementary file 3). Similarly, KEGG GO analysis identified enrichment for two pathways in the differentially expressed 35-gene signature – protein processing in endoplasmic reticulum (*P*=0.02) and regulation of actin cytoskeleton (*P*=0.03) (Figure 3c). Regulation of actin cytoskeleton encompasses signaling to the cytoskeleton through G protein-coupled receptors (GPCRs), integrins (ITGs) and receptor tyrosine kinases (RTKs), leading to diverse cell functions including changes in cell motility, proliferation and survival. This is mediated via a number of signaling cascades, including the MAPK, FAK (focal adhesion kinase) and PI3K (phosphoinositide 3-kinase) pathways.^24, 25, 26, 27^ A schematic representation of the regulation of actin cytoskeleton pathway is shown in Figure 4. A number of genes that were differentially regulated between the sensitive, intermediate and resistant HMCL, including *FGF9*, *F2R*, *ELF3*, *OPN3* (opsin-3), *RGS12* and *KIF4A* (kinesin family member 4A) and are known to be associated with the actin cytoskeleton pathway are also represented in the signaling pathway (Figure 4). A description of these genes and their association with the regulation of actin cytoskeleton pathway components are provided in Supplementary file 4.^28, 29, 30, 31, 32, 33, 34, 35, 36, 37, 38, 39^ As a number of genes known to be associated with the actin cytoskeleton pathway were differentially regulated between the HDACi-sensitive and resistant HMCL, we postulated that the targeting of single genes among those identified would be unlikely to reverse resistance to HDACi, rather it is the global effect of these factors that mediates HDACi resistance. The genes that we identified that are differentially regulated are associated with pathways that are integral to the regulation of actin cytoskeleton rather than being components of the latter. Therefore, we hypothesized that targeting of the identified signaling pathways (PI3K, MEK/ERK and FAK) associated with the differentially expressed genes would mitigate against HDACi resistance (Figure 4).

### Targeting the MEK/ERK, PI3K and FAK pathways overcomes resistance to HDACi in HMCL and primary MM

**Figure 5:**
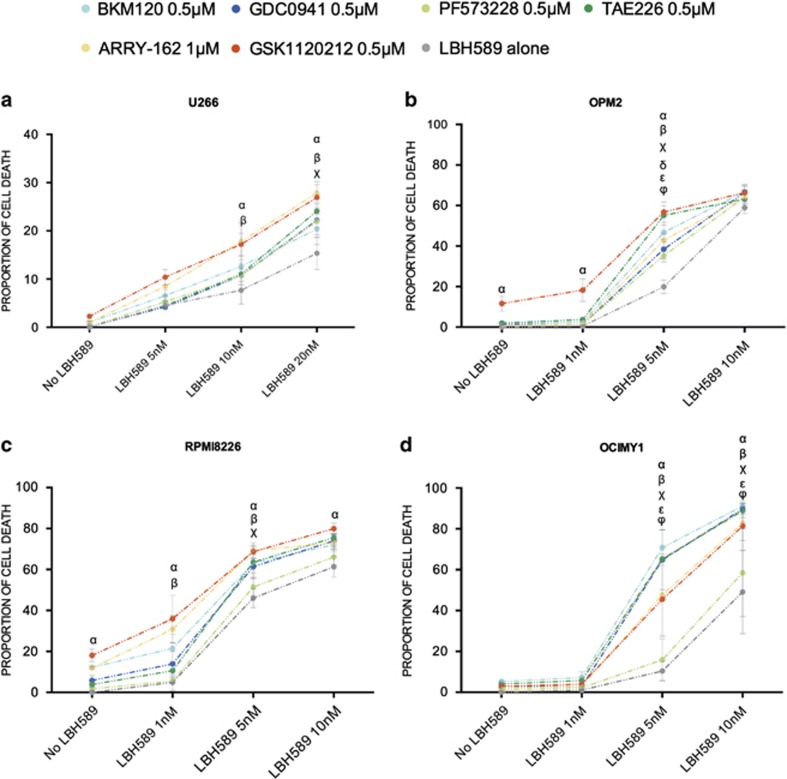
Targeting the actin cytoskeleton overcomes resistance to HDACi in HMCL. Graphs representing proportion of cell death induced in HMCL treated with a combination of HDACi – LBH589 (0, 5, 10 and 20 nM) and MEK/ERK (GSK1120212 or ARRY-162, at 0.5 and 1 *μ*M, respectively), FAK inhibitors (TAE226 and PF50638, both at 0.5 *μ*M) or PI3K inhibitors (GDC-0941 or BKM120, both at 0.5 *μ*M) for 72 h estimated through flow cytometric enumeration of propidium iodide staining. (**a**) HDACi-resistant cell line U266 was treated with inhibitors (LBH589 at 0, 5, 10 and 20 nM) as above and proportion of cell death is shown. (**b**) HDACi-resistant cell line OPM2 was treated with inhibitors (LBH589 at 0, 1, 5 and 10 nM) as above and proportion of cell death is shown. (**c**) HDACi-intermediate cell line RPMI-8226 was treated with inhibitors (LBH589 at 0, 1, 5 and 10 nM) as above and proportion of cell death is shown. (**d**) HDACi-sensitive cell line OCI-MY1 was treated with inhibitors (LBH589 at 0, 1, 5 and 10 nM) as above and proportion of cell death is shown. Each point represents mean±S.E.M. of the proportion of cell death from three individual experiments. Synergistic combinations were determined through two-way ANOVA analysis and significances are as indicated (*α* – *P*<0.05, LBH589 *versus* LBH589+GSK1120212; *β* – *P*<0.05, LBH589 *versus* LBH589+ARRY-162; *χ* – *P*<0.05, LBH589 *versus* LBH589+TAE226; *δ* – *P*<0.05, LBH589 *versus* LBH589+PF50638; *ɛ* – *P*<0.05, LBH589 *versus* LBH589+GDC-0941, *φ* – *P*<0.05, LBH589 *versus* LBH589+BKM120)

To determine if targeting the signaling cascades in the regulation of the actin cytoskeleton pathway would overcome resistance to HDACi, inhibitors to the MAPK, FAK and PI3K pathways were evaluated in combination with LBH589 against HMCL, OCI-MY1, RPMI-8266, OPM2 and U266. The inhibitors chosen are all currently available developmental anti-cancer agents that may progress to use in the clinical setting. A representation of the proportion of cell death induced by the single agents or combination treatment is represented in Figures 5a–d. In HDACi-resistant HMCL (U266 and OPM2), the combinations were found to partially overcome resistance to HDACi (Figures 5a and b) and potentiate cell death in sensitive (OCI-MY1) and intermediate HMCL (RPMI-8226) compared with single agents. It was clear that LBH589 was found to be synergistic with MAPK and FAK inhibitors in all HMCL, with at least one combinations tested (*P*<0.05; Figures 5a–d), whereas with the PI3K/AKT inhibitor synergism was seen only against OPM2 and OCI-MY1 (Figures 5b and d). In U266 and OPM2, the maximal amount of cell death induced with any of the combinations was 30 and 75% respectively; in RPMI-8226 80% and in OCI-MY1 >90%, at the highest concentration of LBH589 utilized, indicating that resistance can be partially overcome in the HDACi-resistant and intermediate HMCL and that as expected the HDACi-sensitive HMCL had maximal cell death induced when this network of pathways was targeted in combination with HDACi.

**Figure 6:**
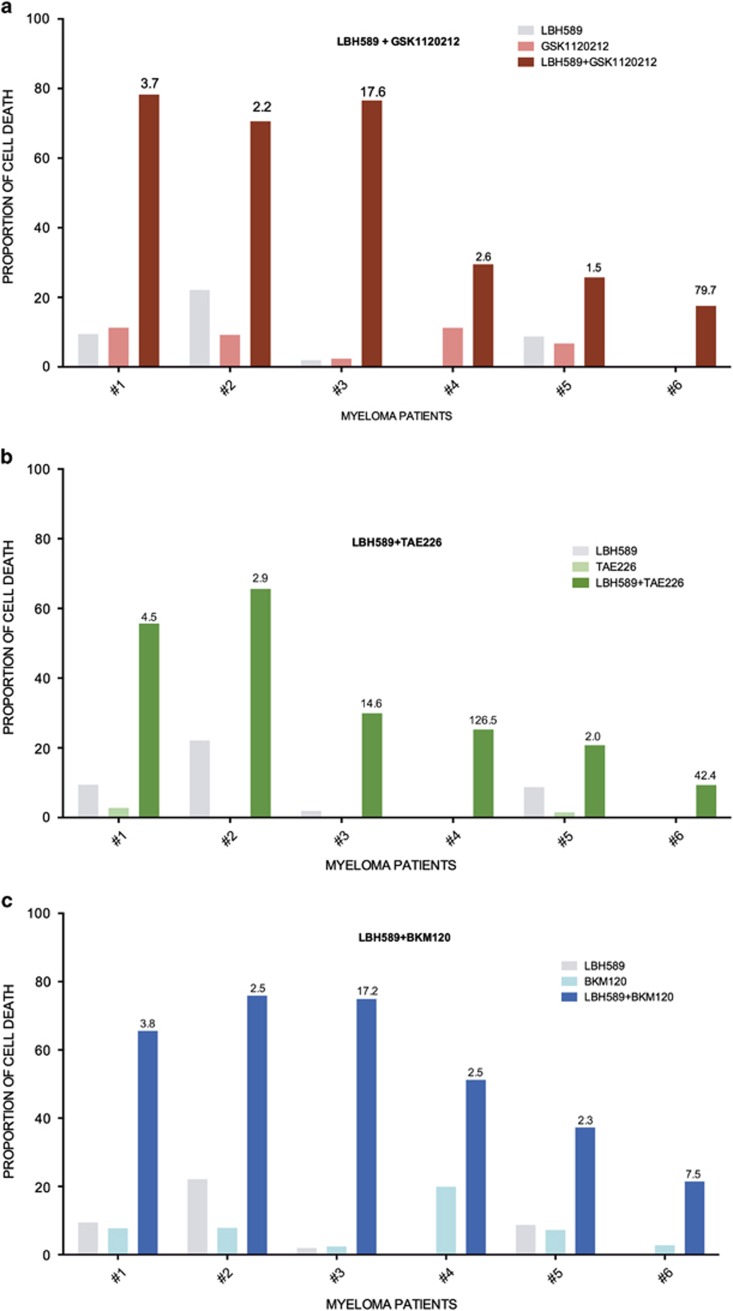
Targeting the actin cytoskeleton overcomes resistance to HDACi in primary MM. (**a**) Graphs representing the proportion of cell death induced in MM patients (*n*=6) treated with HDACi – LBH589 (5 nM), GSK1120212 (0.5 *μ*M) and combination. (**b**) Graphs representing the proportion of cell death induced in MM patients (*n*=6) treated LBH589 (5 nM), TAE226 (0.5 *μ*M) and combination. (**c**) Graphs representing the proportion of cell death induced in MM patients (*n*=6) treated with LBH589 (5 nM), BKM120 (0.5 *μ*M) and combination for 72 h. Cell death was estimated through flow cytometric enumeration of APO2.7 staining in CD38+ and CD45− MM cells. Numbers on top of the bars representing cell death proportion in the drug combination samples represent the SQ of the stated combinations. SQ of >1 represents a synergistic combination

For the primary MM tumor treatment (*n*=6 for each combination), the MAPK inhibitor GSK1120212, FAK inhibitor TAE226 and PI3K inhibitor BKM120 were chosen based on the data generated from HMCL. Cell death induced with 5 nM of LBH589 and 0.5 *μ*M of each partner drug is shown (Figures 6a–c). In all patients tested, GSK1120212, TAE226 and BKM120 were able to induce synergistic cell death in combination with LBH589 validating that resistance to HDACi can be overcome by targeting actin cytoskeleton related signaling cascades. In all assays, the proportion of death induced in non-MM compartment was also assessed and ranged between 4 and 25% confirming the existence of a potential therapeutic window for the combinations evaluated. On the basis of these data presently available developmental targeted therapies aimed at key pathways of the actin cytoskeleton could therefore be potentially utilized in combination with HDACi for MM therapy.

## Discussion

HDACi have been shown to have efficacy against a range of hematological tumors, however, in MM, HDACi have demonstrated only limited single agent activity and it is clear that the full therapeutic potential of HDACi will only be realized through their rational combination with other anti-cancer agents. Although PIs have emerged as promising combination partners for HDACi and findings from recent clinical trials combining vorinostat with bortezomib suggest that a subset of MM patients with advanced disease do benefit from the combination, the demonstration of benefit in unselected less heavily treated patients remains problematic. Therefore, it is critical to identify biomarkers of responsiveness to HDACi to enable more selected patient approaches and to identify rational drug combinations to overcome resistance to HDACi. In this report, using a microarray-based strategy, we provide evidence that dysregulation of key pathways is associated with resistance to HDACi and that this resistance may be overcome with appropriate drug combination approaches. This approach has been specifically designed to identify new partner drugs that can be utilized in MM therapeutics.

Initial GEP analyses revealed that differentially regulated probes between the sensitive and resistant cell lines (*n*=97 probes) were enriched for potentially seven KEGG pathways. However, it should be acknowledged that some pathways may be over-represented in the analysis as some genes, including *PI3KCG* (phosphatidylinositol 3-Kinase p110 gamma) and *FGF9*, are components of more than one pathway (Supplementary file 3). Therefore, a more stringent and independent analysis was undertaken to correlate GEP with increasing levels of HDACi resistance. Despite the primary translocations and secondary genetic lesion these HMCL harbor, it was striking to identify a unique 35-gene signature. Among the two pathways enriched for in the differentially regulated gene set, the regulation of the actin cytoskeleton pathway was of interest. This pathway represents a major network of proteins that orchestrate motility, invasion, survival and growth of normal cells, and is often dysregulated in tumor cells.^40^ A number of genes associated with the actin cytoskeleton pathway were differentially regulated between the sample groups and are known to act through the MAPK, FAK and PI3K pathways (Figure 4, Supplementary file 4). Subsequently, combination of HDACi with agents directed against these pathways in HMCL and primary MM confirmed synergistic cell death compared with single agent treatment. Indeed, the MEK/ERK signaling pathway has been suggested to be associated with resistance to HDACi both in solid tumors and hematological malignancies.^7, 8, 41, 42^ However, these studies address acquired resistance to HDACi following exposure to chemotherapeutic agents, rather than inherent resistance to HDACi. In MM, aberrant MEK/ERK signaling is known to have an important role in disease progression and prognosis and small molecule inhibitors have shown promise in pre-clinical evaluation.^43, 44, 45^ Frequent activation of the PI3K/AKT pathway in MM cells has also been observed^46, 47^ and a number of PI3K inhibitors induce apoptosis in MM cells.^48, 49, 50, 51^ Moreover, the combination of PI3K inhibition and HDACi has demonstrated synergistic killing in some cancers,^52, 53, 54^ however, the rational utilization of this combination for MM has not been reported elsewhere. Similarly, abnormal expression of FAK is correlated with proliferation of MM cells and disease progression^55, 56^ but only limited data on FAK inhibition in MM is available and to our knowledge the utilization of FAK inhibition in combination with HDACi has not been previously explored. Therefore, utilization of available targeted therapies aimed at key pathways integral to the actin cytoskeleton in combination with HDACi warrants further evaluation.

The molecular mechanisms of resistance to HDACi are probably a combination of dysregulated pathways with no one gene responsible for inherent resistance to HDACi. Likewise, the mechanism of synergism observed with partner drugs could be multi-factorial owing to the heterogeneous nature of the disease and translocations/mutations that HMCL and MM samples harbor. It should also be noted that within the two HDACi-resistant HMCL, although both cell lines were sensitive to HDACi and MEKi combination, OPM2 was more sensitive to HDACi and FAKi and PI3K combinations. Likewise, in primary MM, although all classes of inhibitors showed synergistic cell death with LBH589, there was clearly inter-patient variability in response to the specific inhibitors presumably dictated by their genetic heterogeneity. Importantly the synergistic activity of the combinations when tested against primary MM cells in an autologous narrow co-culture assay provides evidence that bone marrow microenvironment-related factors contributing to MM resistance are also overcome. Although all primary MM tested confirmed that the combinations are synergistic, it would be vital to validate the HDACi resistance genetic signature in a larger primary tumor sample set and this will inform our future investigations.

Although targeting the actin cytoskeleton proved to be an effective combination strategy, our data also provide a rationale for the exploratory analysis of other strategies that may overcome MM resistance to HDACi. For instance, FBXO6 and E3 ubiquitin ligase, which was downregulated in the resistant HMCL, may also contribute to HDACi resistance *via* the modulation of checkpoint kinase I (CHK1). FBXO6 targets activated CHK1 for degradation^57^ and over-expression of CHK1 has been shown to contribute to HDACi resistance.^58^ Therefore, one could postulate that the absence of FBXO6 with subsequent impaired clearance of activated CHK1 and resulting inhibition of apoptosis may identify MM tumors where CHK1 inhibitors could be successfully combined with HDACi. Furthermore, within the 35-probe set, a number of genes known to have a role in oncogenesis, including *POU4F1* (POU domain, class 4, transcription factor 1)^59^ and *CHFR*,^60^ were identified, but are not presently drugable.

HDACi have emerged as one of the most promising classes of epigenetic modifying agents for cancer treatment and the identification of rational choices for combination therapies, that will be clearly context-dependent, based on the type of cancer and available anti-cancer agents is critical for their further therapeutic development. Until now a comprehensive determination of the factors associated with resistance to HDACi in MM has not been undertaken and this report provides the first evidence of pathways associated with HDACi-resistance in MM using a novel genomic approach. The combination of HDACi and actin cytoskeleton pathway inhibitors results in synergistic cytotoxic effect against MM cells and thus has significant clinical implications. Moreover, based on our identification of this 35-gene signature we are presently determining whether it will provide a basis for the validation of biomarkers predictive of HDACi response *in vivo,* thus affording the selection of MM patient populations that are better suited to receiving HDACi therapy.

## Materials and Methods

### Reagents

The HDACi (LBH589, SAHA and FK228) were purchased from Selleck Chemicals, Munich, Germany. The MAPK inhibitors, GSK1120212 and ARRY-162, were obtained from Selleck Chemicals and AbMole BioScience, HongKong, China, respectively. FAK inhibitor TAE226 was from Selleck Chemicals and PF573228 was from Tocris Bioscience, Bristol, UK. Phosphatidylinositol 3-kinases (PI3K) inhibitors GDC-0941 and BKM120 were obtained from Selleck Chemicals. All compounds were dissolved in dimethyl sulfoxide and stock drug solutions were diluted in complete RPMI-1640 culture medium (Life Technologies, Mulgrave, VIC, Australia) to various concentrations for experimentation.

### Human myeloma cell lines

The HMCL U266, NCI-H929, LP-1 and RPMI-8226 were obtained from the American Type Culture Collection (Rockville, MD, USA). OPM2 was obtained from Deutshe SammLung von Mikro-orgaanismen und Zellculturen (Braunshwieig, Germany). KMS11 was a kind gift from Dr. Takemi Otsuki, Kawasaki Medical School, Japan. ANBL-6, XG-1 and OCI-MY1 were kind gifts from Winthrop P Rockefeller Cancer Institute (Little Rock, AR, USA). HMCL were grown and treated at a density of 2.0  ×  10^5^ cells/ml in RPMI-1640 media supplemented with 10% heat inactivated fetal bovine serum (Lonza, Mt Waverley, VIC, Australia) and 2 mM L-glutamine (Life Technologies).

### Estimation of cell death proportion following treatment of HMCL

To determine the response of HMCL (*n*=9) to HDACi, cells were treated with HDACi (LBH589 and FK228 at 10, 50 and 100 nM and SAHA at 1, 5 and 10 *μ*M) for 48 h (each *n*=3). For HDACi combination experiments, OPM2, OCI-MY1, RPMI-8226 and U266, were treated simultaneously with MAPK, FAK or PI3K inhibitors (0.5 and 1 *μ*M) in combination with LBH589 (1, 5, 10 and 20 nM) for 72 h (*n*=3). Following treatment, the cells were harvested and washed with 1 × phosphate-buffered saline (PBS) before staining with propidium iodide (Sigma-Aldrich, Castle Hill, NSW, Australia; 60 ng/ml in PBS). Samples were acquired on a FACSCaliber Flow Cytometer (Becton Dickinson, San Jose, CA, USA) and analysed on FlowJo V7.6.2 (Treestar, Ashland, OR, USA). The proportion of propidium iodide-positive cells was quantitated by subtracting the background death in untreated cells. GraphPad Prism V5.0d was utilized to generate graphs and determine statistical significance.

### Treatment of primary MM with HDACi and partner drugs

Primary MM samples were obtained from previously treated MM patients at the time of disease relapse following written informed consent. Briefly, bone marrow aspirates were collected, centrifuged at 300 × *g* for 10 min at room temperature, the supernatant was then removed. The cell pellet was resuspended in 1 × PBS to three times the original sample volume. Ficoll-Paque Plus (GE Healthcare Life Sciences, Rydalmere, NSW, Australia) was underlaid using a cannula at a ratio of 1:2 to the sample. The BM sample was then centrifuged without the brake at 800 g for 25 min at room temperature. The mononuclear cell layer was then collected and transferred to a new tube and washed in PBS. The red blood cells were then lysed with NH_4_Cl (8.29 g/l ammonium chloride, 0.037 g/l EDTA, 1 g/l potassium bicarbonate) solution and incubated at 37 °C for 5 min. The cells were then washed in PBS, and quantitated by hemocytometer. Primary samples were then cultured in complete RPMI-1640 media with LBH589 (1 and 5 nM) and GSK1120212/TAE226/BKM120 (at 0.5 and 1 *μ*M) for 72 h (*n*=6 patients per combination). Samples were stained for CD45-FITC (fluorescein isothiocyanate) (BD, North Ryde, NSW, Australia), CD38-PerCP-Cy5.5 (peridinin chlorophyll–cynanine5.5) (BD) and CD138-PE (R-Phycoerythrin) (BD) to identify MM population (CD38+, CD138+, CD45+/−) and analyzed by flow cytometry. Drug-induced MM-specific cell apoptosis (CD38+ and CD45−/+) was then compared with untreated and vehicle controls by staining for CD45-FITC, CD38-PerCP-Cy5.5 (BD) and Apo 2.7-PE (Immunotech Beckman Coulter, Mt Waverley, VIC, Australia). Proportion of cell death induced in non-MM cells was also determined (CD138−, CD38−, APO2.7+). Single drug-treated cells were compared with combination-treated cells, and the presence of synergism demonstrated by the derivation of the Synergism quotient (SQ) for each combination, where the SQ is the net effect of the combination [drug A+drug B] divided by the sum of the net individual effect [A]+[B]. Any SQ greater than 1 thus indicates a synergistic effect.

### Microarray analysis

Total RNA from HMCL was prepared using RNeasy kit (QIAGEN, Doncaster, VIC, Australia) and any residual genomic DNA was removed utilizing the Turbo-DNase I kit (AMBION, Austin, TX, USA). The quality and quantity of the RNA obtained was assessed using Nanodrop 2000 Spectrophotometer and Quant-IT (Life Technologies), respectively. Samples were assessed on the Illumina HT-12 V2 platform. Raw signal intensity data from Illumina HT-12 slides (www.illumina.com) was subjected to variance stabilization transformation including background correction. Each expression value below 50 was adjusted to 50, which was approximately 50% of the background noise level. Hereafter, signal intensities were log2 transformed and quantile normalized. Probes with variance smaller 0.5 were excluded from the subsequent analysis. Unsupervised clustering analysis was performed to identify differential expression between the categories (resistant, intermediate and sensitive). Analysis of variance (ANOVA) analysis of normalized probe intensities values was performed in Partek Genomic SuiteTM software, version 6.5 (Partek Inc., St. Louis, MO, USA). ANOVA was used to calculate significance of variation in normalized expression values between sample groups; the fold change of gene expression was calculated as a mean ratio. Probes with an unadjusted *p*-value of 0.05 or less (no False Discovery Rate correction was applied) and an absolute fold change of 1.5 or more were defined as differentially expressed. GO ANOVA and GO enrichment analysis were performed on the differentially expressed probes in Partek Genomics Suite 6.5, using the KEGG GO database (www.genome.jp/kegg/). Enrichment Fisher exact *P*-value was calculated based on the number of genes in the provided gene list in relation to the number of genes in gene groups in the genome annotation file and the enrichment score derived from the negative antilog of that *P*-value. If a functional group has an enrichment score over 1, the functional category is defined as overexpressed. A value of three corresponds to significant over expression (*P*-value of <0.05). The larger the score, the more differentially expressed the genes in the group.

### Quantitative RT-PCR

Reverse transcription was performed on 200 ng of total RNA with 100 U of Superscript III reverse transcriptase (Life Technologies) and random hexamers (Life Technologies, 2.5 *μ*M final concentration), according to manufacturers guidelines. Quantitative RT-PCR was performed for genes shortlisted from the microarray results. The reaction mixture consisted of 8 *μ*l of Power-SYBR PCR Master Mix (Life Technologies) with 500 nM of each forward (F) and reverse (R) primers for target genes and 2 *μ*l of diluted template cDNA. The list of primers used is presented in Supplementary file 1. PCR was performed with a 7900HT real-time system (Life Technologies) at 95 °C for 10 min, with 45 cycles of amplification 95 °C for 15 s, 62 °C for 30 s, 72 °C for 30 s. Each reaction was performed in triplicate. For sample loading normalization, b-actin (*ACTB*) (encoding *β*-actin) was used and the mRNA levels of *ACTB* were not significantly different between samples. Amplified products were all verified by melting curves analysis and agarose gel electrophoresis. Data were analyzed using SDS software version 2.3 (Life Technologies) and copy number of target genes was determined by the comparative threshold cycle method (ΔΔCT) using the Pfaffl method. Data are presented as mean±S.E.M.

## Abbreviations

ACTB: b-actin
ANOVA: analysis of variance
Bcl-2: B-cell lymphoma 2
BIRC2/3: baculoviral IAP repeat-containing protein 2/3
BRAF: v-Raf murine sarcoma viral oncogene homolog B1
CDKN2C: cyclin-dependent kinase inhibitor 2C
CHK1: checkpoint kinase 1
ELF3: E74-like factor 3
ERK: extracellular signal regulated kinases
F2R: coagulation factor II receptor
FAK: focal adhesion kinase
FACS: fluorescence activated cell sorting
FBXO6: F-box protein 6
FGF9: fibroblast growth factor 9
FGFR3: fibroblast growth factor receptor 3
FITC: fluorescein isothiocyanate
GEP: gene expression profiling
GO: gene ontology
GPCR: G protein-coupled receptors
GSTO1: glutathione S-transferase omega-1
HDACi: histone deacetylase inhibitors
HMCL: human myeloma cell lines
HSP90: heat shock protein 90
IL12A: interleukin 12A
ITG: integrins
JAK-STAT: janus kinase/signal transducers and activators of transcription
KIF4A: kinesin family member 4A
KRAS: v-Ki-ras2 Kirsten rat sarcoma viral oncogene homolog
MAPK: mitogen-activated protein kinase
MM: multiple myeloma
mTOR: mammalian target of rapamycin
NF-KB: nuclear factor kappa-light-chain-enhancer of activated B cells
NH_4_Cl: ammonium chloride
NRAS: neuroblastoma RAS viral oncogene homolog
OPN3: opsin-3
PBS: phosphate-buffered saline
PCA: principle component analysis
PE: R-Phycoerythrin
PerCP-Cy5.5: peridinin chlorophyll–cynanine5.5
PI3K: phosphoinositide 3-kinase
PI3KCG: phosphatidylinositol 3-Kinase p110 gamma
POU4F1: POU domain, class 4, transcription factor 1
PSEN2: presenilin 2
RB1: retinoblastoma protein
RGS12: regulator of G-protein signaling 12
RTK: receptor tyrosine kinases
RT-PCR: reverse transcriptase-polymerase chain reaction
SQ: synergy quotient
TRAIL: TNF-related apoptosis-inducing ligand
TP53: phosphoprotein p53

## Supplementary Material

Supplemental File 1

Supplemental File 2A

Supplemental File 2B
