# Supplementary figures and images for: Overcoming inherent resistance to histone deacetylase inhibitors in multiple myeloma cells by targeting pathways integral to the actin cytoskeleton
Source: Cell Death Dis. 2014 Mar 20;5(3):e1134–. doi: 10.1038/cddis.2014.98 (PMC3973216; doi:10.1038/cddis.2014.98)

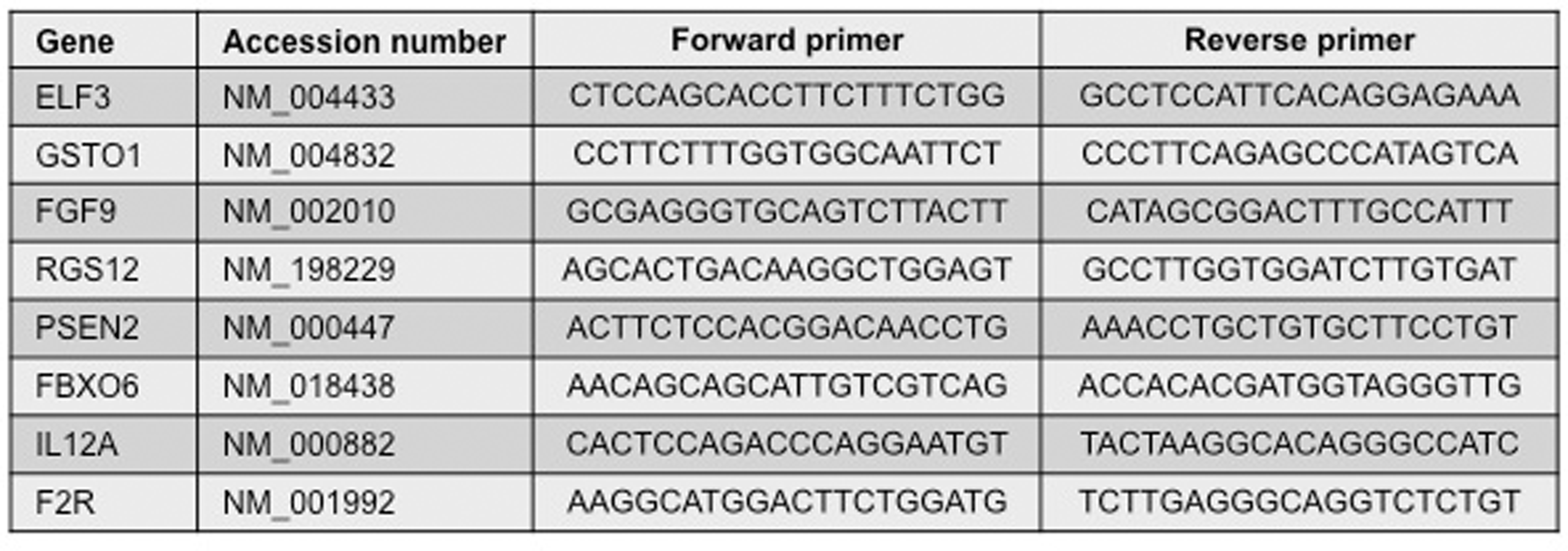

Supplement: Supplemental File 1 [file cddis201498x1.tif]

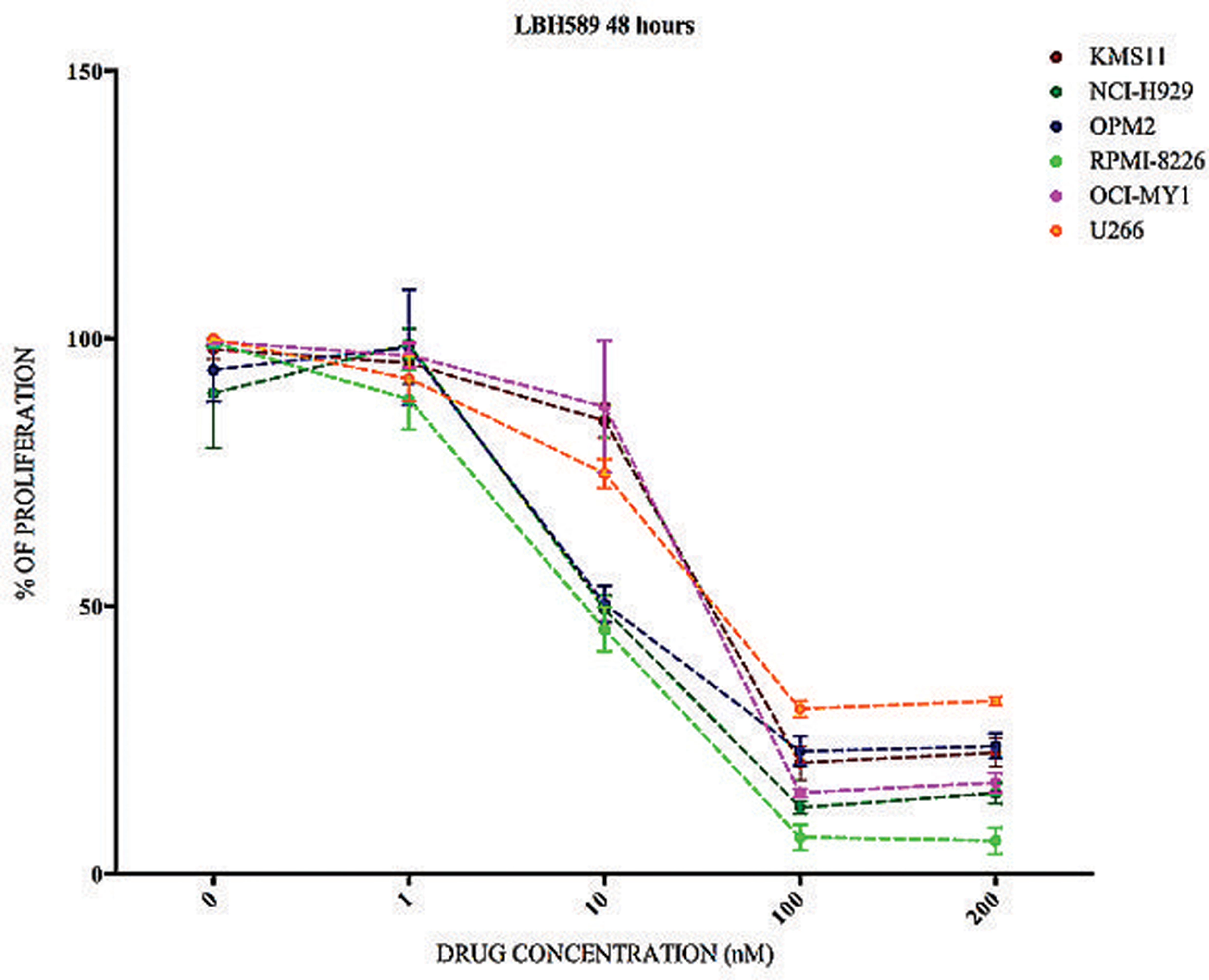

Supplement: Supplemental File 2A [file cddis201498x2.tif]

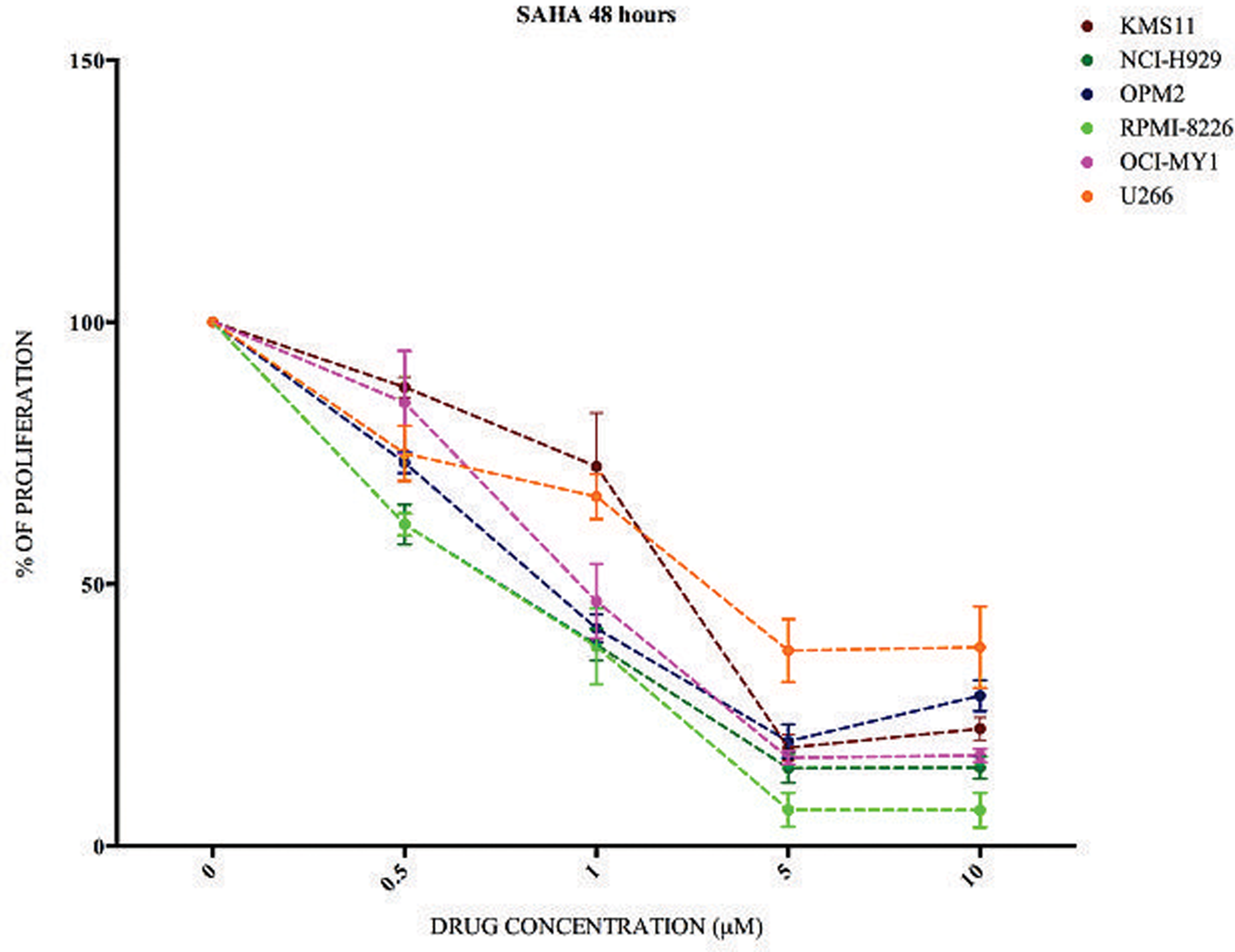

Supplement: Supplemental File 2B [file cddis201498x3.tif]
