# Supplementary material for: Overcoming inherent resistance to histone deacetylase inhibitors in multiple myeloma cells by targeting pathways integral to the actin cytoskeleton
Source: Cell Death Dis. 2014 Mar 20;5(3):e1134–. doi: 10.1038/cddis.2014.98 (PMC3973216; doi:10.1038/cddis.2014.98)
Supplement: Supplementary Information [file cddis201498x6.doc]

**Supplemental file 1: List of oligonucleotide sequences**

Table shows list of genes, accession numbers and corresponding oligonucleotide sequences designed for amplification using quantitative real-time PCR.

**Supplemental file 2A: Inhibition in proliferation following treatment with LBH589 at 48 hours in HMCL**

Graph represents the proportion of proliferation inhibition in six HMCL following treatment with 0, 1, 10, 100 and 200nM of LBH589 at 48 hours. MTS assays (Cell Titer 96 Aqueous One Solution Cell Proliferation Assay, Promega) were used to quantify the percentage of metabolically active cells.

**Supplemental file 2B: Inhibition in proliferation following SAHA treatment at 48 hours in HMCL**

Graph represents the proportion of proliferation inhibition in six HMCL following treatment with 0, 0.5, 1, 5 and 10 μM of SAHA at 48 hours assessed using the MTS assay.

**Supplemental file 3: Differentially regulated gene sets and pathway analysis**

Excel file provides the differentially regulated probes between HDACi-sensitive and resistant HMCL (n=97) and probes that had >80% correlation to grade of sensitivity (n=35). Gene ontology ANOVA and gene ontology enrichment analysis results for both sets (n=97 and n=35) are also provided.

**Supplemental file 4: List of differentially regulated genes associated with the regulation of actin cytoskeleton pathway**

Excel file provides a description of the differentially regulated genes and their association with the regulation of actin cytoskeleton pathway components.
